# The characteristics of mitral regurgitation: Data from patients admitted following acute myocardial infarction

**DOI:** 10.1016/j.dib.2021.107451

**Published:** 2021-10-12

**Authors:** Harish Sharma, Ashwin Radhakrishnan, Peter Nightingale, Samuel Brown, John May, Kieran O'Connor, Iqra Shakeel, Nawal Zia, Sagar N. Doshi, Jonathan N. Townend, Saul G. Myerson, Paulus Kirchhof, Peter F. Ludman, M. Adnan Nadir, Richard P. Steeds

**Affiliations:** a Institute of Cardiovascular Sciences, University of Birmingham, Birmingham, UK; b Department of Cardiology, University Hospitals Birmingham, Birmingham, UK; c Institute of Translational Medicine, University Hospitals Birmingham, Birmingham, UK; d Medical School, College of Medical and Dental Sciences, University of Birmingham, UK; e Division of Cardiovascular Medicine, Radcliffe Department of Medicine, University of Oxford, Oxford, UK; f Department of Cardiology, University Heart and Vascular Center UKE Hamburg, Germany; g German Center for Cardiovascular Research (DZHK), Partner Site Hamburg/Kiel/Lübeck, Germany

**Keywords:** Mitral regurgitation, Secondary MR, Myocardial infarction, Valvular disease

## Abstract

Data were collected on patients admitted to the Queen Elizabeth Hospital Birmingham with type-1 myocardial infarction during 2016 and 2017 inclusively, who were treated by percutaneous intervention and had pre-discharge transthoracic echocardiography. The data were obtained from prospectively maintained hospital databases and records. Echocardiography was performed and reported contemporaneously by accredited echocardiographers. The purpose was to understand the prevalence and characteristics of mitral regurgitation (MR) after acute MI, including patients with ST-elevation (STEMI) and non-ST elevation MI (NSTEMI). MR was observed in 294/1000 patients with the following relative severities: mild = 76%, moderate = 21%, severe = 3% [1]. MR was graded by multiparametric quantification including proximal isolvelocity surface area (PISA), vena contracta (VC), effective regurgitant orifice area (EROA) and regurgitant volume (RVol). Amongst all patients with MR (n=294), PISA was performed in 89/294 (30%), VC 75/294 (26%), EROA in 53/294 (18%) and RVol in 26/294 (9%). Amongst patients with moderate or severe MR (n=70), PISA was performed in 57/70 (81%), VC in 55/70 (79%), EROA in 46/70 (66%) and RVol in 25/70 (36%). Characteristics of MR following acute MI were also assessed including frequency of reported leaflet thickness (259/294 = 88%) and mitral annular calcification (102/294 = 35%). Furthermore, the effect of MI on pre-existing MR was investigated and patients with pre-existing MR who continue to have MR after acute MI were found to have progression of MR by one grade in approximately 25% of cases. Finally, using Cox proportional hazards univariate analysis, significant factors associated with mortality in patients with MR post-MI include age (HR 1.065; 95% CI 1.035-1.096; p<0.001), creatinine clearance, (HR 0.981; 95% CI 0.971-0.991; p<0.001), left ventricular ejection fraction (LVEF) (HR 0.966; 95% CI 0.948-0.984; p<0.001), indexed left ventricular end-diastolic volume (LVEDVi) (HR 1.016; 95% CI 1.003-1.029; p=0.018), indexed left ventricular end-systolic volume (LVESVi) (HR 1.021; 95% CI 1.008-1.034; p=0.001), indexed left atrial volume (HR 1.026; 95% CI 1.012-1.039; p<0.001), and those with intermediate likelihood of pulmonary hypertension (pHTN) (HR 2.223; 95% CI 1.126-4.390; p=0.021); or high likelihood of pHTN (HR 5.626; 95% CI 2.189-14.461; p<0.001). Age and LVEF were found to be independent predictors of mortality on multivariate analysis [1].

## Specifications Table

**Table:** 

Subject	Cardiology and Cardiovascular Medicine
Specific subject area	Valvular Heart Disease
Type of data	Table Chart Graph
How data were acquired	Retrospective search of prospectively maintained cardiac catheterization laboratory together with the hospital records of patients fulfilling the inclusion criteria.
Data format	Analysed
Parameters for data collection	Patients admitted with type-1 myocardial infarction treated between Jan 1^st^ 2016 and Dec 31^st^ 2017, treated by percutaneous coronary intervention (PCI) and with pre-discharge transthoracic echocardiography
Description of data collection	Prospectively maintained hospital databases were searched for patients fulfilling the inclusion criteria. One thousand consecutive patients’ hospital records were examined to identify clinical data pertaining to demographics, risk factors, presentation and past history.
Data source location	Institution: Queen Elizabeth Hospital Birmingham City/Town/Region: Birmingham, West Midlands Country: United Kingdom
Data accessibility	With the article
Related research article	H. Sharma, A. Radhakrishnan, P. Nightingale, *et al.* Mitral regurgitation following acute myocardial infarction treated by percutaneous coronary intervention – prevalence, risk factors and predictors of outcome, American Journal of Cardiology, In Press.

## Value of the Data

• These data are important because they characterise mitral regurgitation observed after acute MI. In particular, these data show that a significant proportion of patients have posteriorly eccentric jets which may explain why the use of multiparametric quantification in real world analysis of patients with MR is not ubiquitous.
• This data is useful to cardiologists, echocardiographers and those shaping international guidelines, particularly as echocardiography is currently recommended as the gold standard method of assessing mitral regurgitation after acute MI.
• Currently, international guidelines recommend multiparametric assessment of MR using echocardiography. These data show that despite best efforts, the recommended parameters are not universally measured in the real world and thus there may be a greater role for other imaging modalities e.g. cardiovascular magnetic resonance imaging.
• These data also show that the prevalence and relative severities of MR in a combined population of Non-ST elevation and ST-elevation MI (STEMI) patients is similar to those found in a large prospective study of STEMI patients only.
• Patients with pre-existing MR who continue to have MR after acute MI develop worsening of MR by one grade in approximately 25% of cases.

## 1 Data Description

**Fig. 1:**
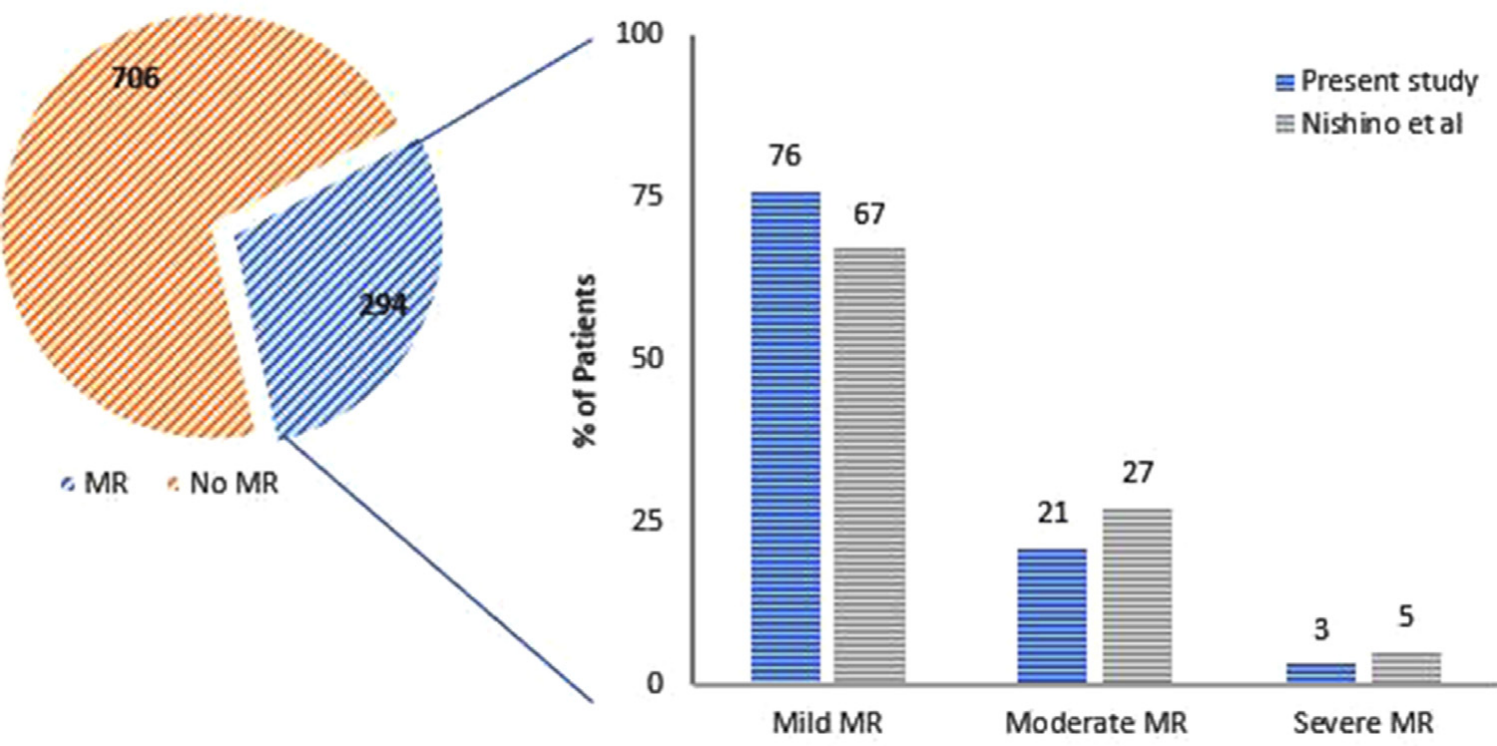
Relative MR severity of the study population in relation to MR severity seen by Nishino et al. in the NSTEMI population [2].

Fig. 1 – pie chart demonstrates the proportions of patients with (294/1000) and without (706/1000) mitral regurgitation (MR) following acute myocardial infarction (MI). The bar chart shows the relative MR severities in this study which included patients with ST-elevation MI (STEMI) and non-ST elevation MI (mild = 76%, moderate = 21%, severe = 3%), in relation to a similar study by Nishino *et al*. [2] which studied a STEMI population only (mild = 67%, moderate = 27%, severe = 5%).

**Fig. 2:**
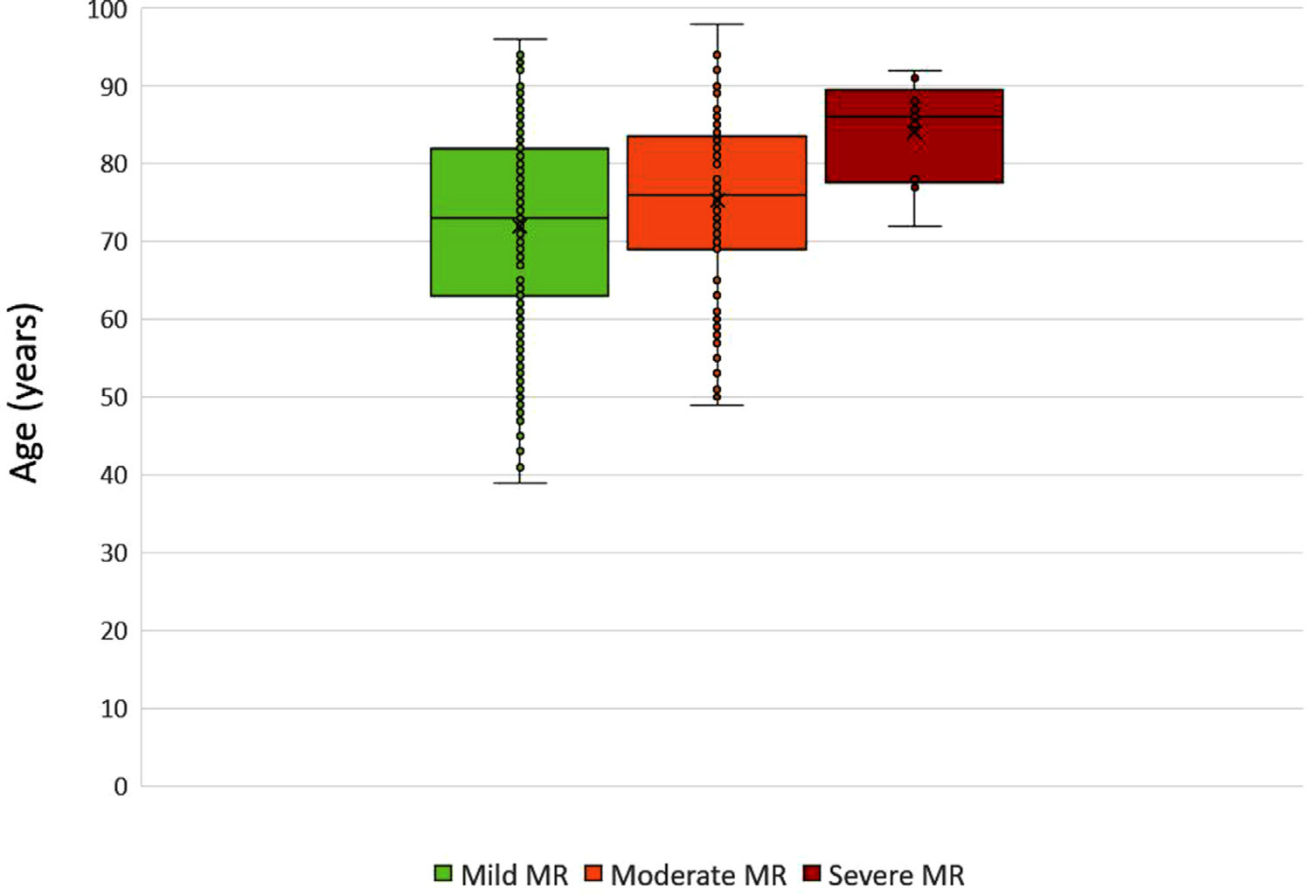
Box and whisker plots demonstrating the association between age and severity of MR.

Fig. 2 – Box and whisker plots demonstrating the association between age and severity of MR. Patients with mild MR had a median age of 73, interquartile range of 63-82 years with minimum and maximum ages of 39 and 96 respectively. Patients with moderate MR had a median age of 76, interquartile range of 69-84 years, with minimum and maximum ages of 49 and 98. Patients with severe MR had a median age of 86, interquartile range of 78-90 years, with minimum and maximum ages of 72 and 92.

**Fig. 3:**
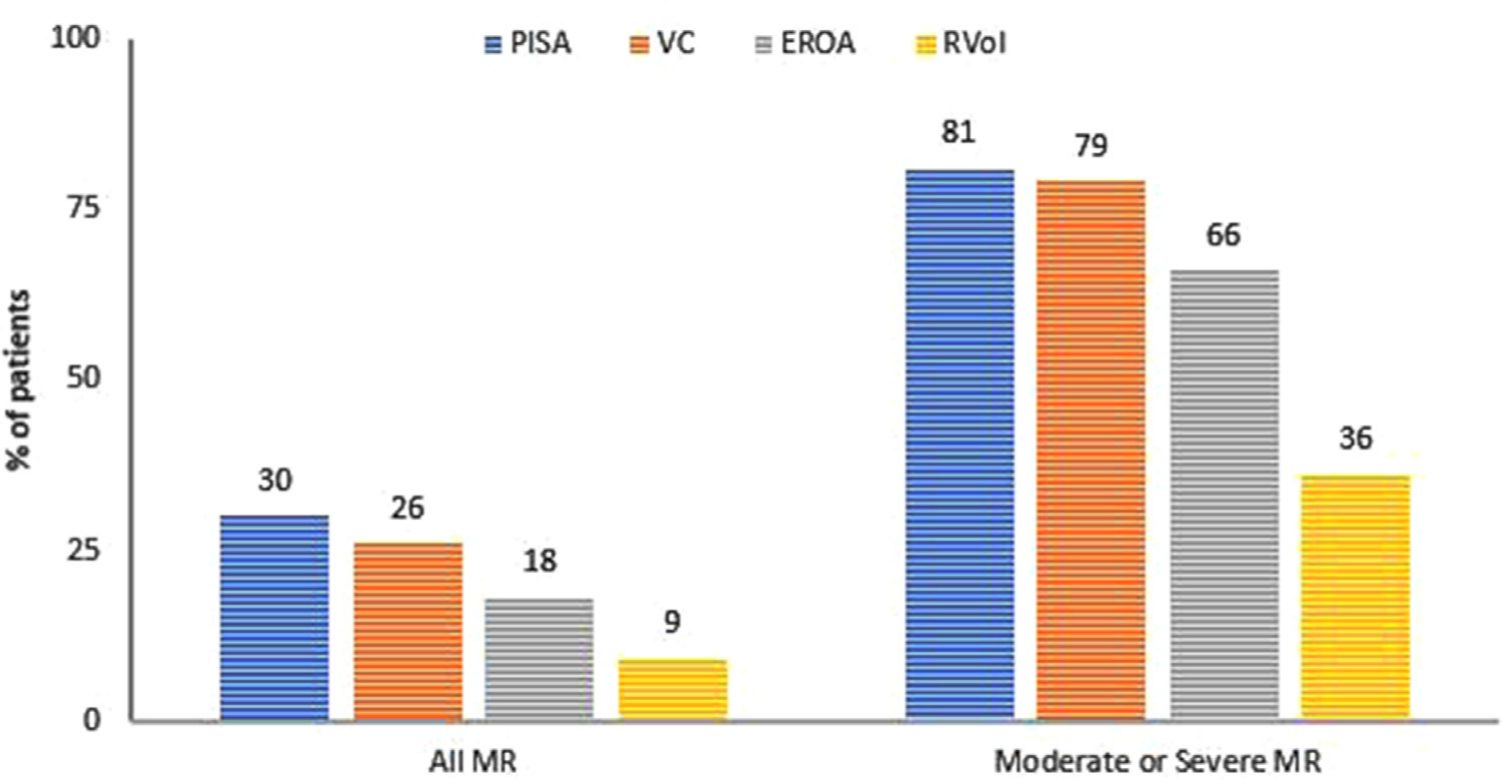
proportions of patients assessed by PISA, EROA, VC and RVol.

Fig. 3 – bar chart demonstrates the proportions of patients with MR assessed by proximal isovelocity surface area (PISA), vena contracta (VC), effective regurgitant orifice area (EROA) and regurgitant volume (RVol). Amongst all patients with MR (n=294), PISA was performed in 89/294 (30%), VC 75/294 (26%), EROA in 53/294 (18%) and RVol in 26/294 (9%). Amongst patients with moderate or severe MR (n=70), PISA was performed in 57/70 (81%), VC in 55/70 (79%), EROA in 46/70 (66%) and RVol in 25/70 (36%).

**Fig. 4:**
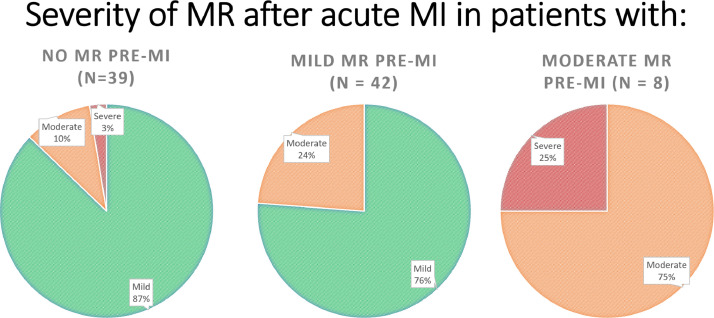
chart demonstrating MR severity following acute MI in patients with and without pre-existing MR.

Fig. 4 – pie charts demonstrating the severity of MR after acute MI in patients with and without pre-existing MR. In patients without prior MR (n=39) but who develop MR post-MI, proportions of MR severity are as follows: 34/39 (87%) mild MR, 4/39 (10%) moderate MR, 1/39 (3%) severe MR. In patients with pre-existing mild MR (n=42) and who continue to have MR post-MI, MR severity worsened by one grade to moderate in 10/42 (24%) and remained mild in 32/42 (76%). In patients with pre-existing moderate MR (n=8) and who continue to have MR post-MI, MR severity worsened by one grade to severe in 2/8 (25%) and remained moderate in 6/8 (75%).

**Fig. 5:**
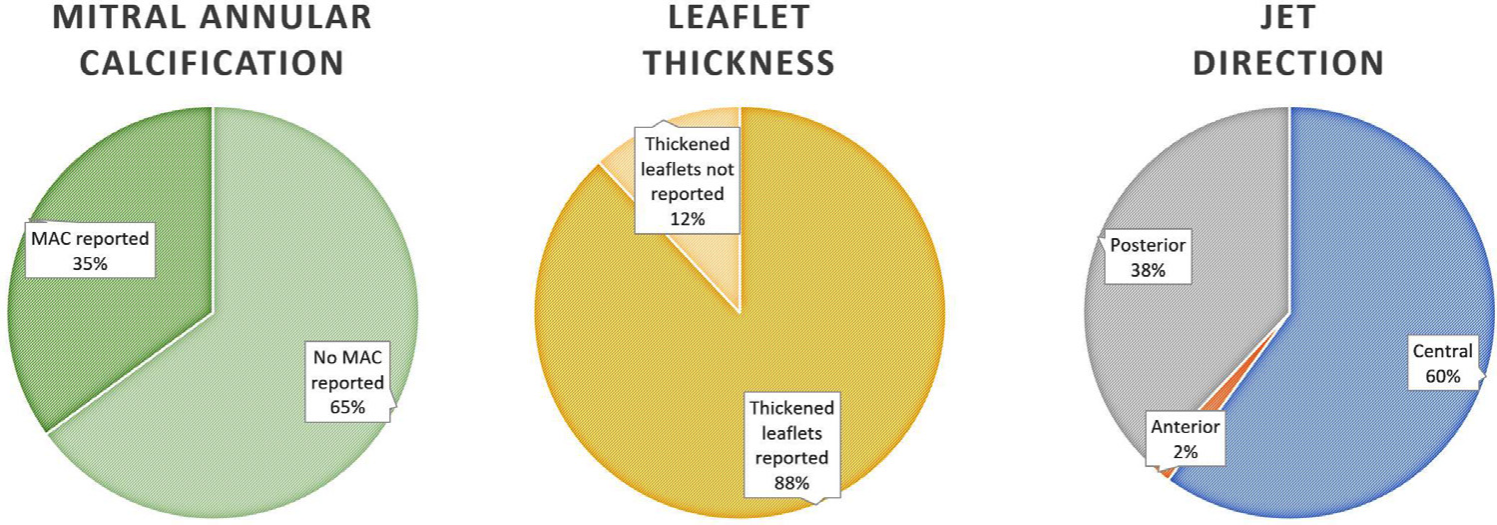
chart demonstrating proportions of patients with reported mitral annular calcification, leaflet thickening and regurgitant jet direction.

Fig. 5 – pie charts demonstrating the proportion of MR patients with reported mitral annular calcification (102/294 = 35%) versus no reported mitral annular calcification (191/294 = 65%), thickened leaflets (259/294 = 88%) versus no reported leaflet thickening (35/294 = 12%), and mitral regurgitant jet direction [central = 176/294 (60%), posterior = 112/294 (38%), anterior = 6/294 (2%)] Table 1.

**Table 1:** Univariate risk factors for mortality.

Variable	Hazard Ratio	95% CI	P Value
Age	1.064	1.035 – 1.096	< 0.001 ⁎⁎
Hypertension	0.583	0.315 – 1.082	0.083
Diabetes	1.315	0.738 – 2.344	0.353
Creatinine Clearance	0.981	0.971 – 0.991	< 0.001 ⁎⁎
LVEF	0.966	0.948 – 0.984	< 0.001 ⁎⁎
LVEDVi	1.016	1.003 – 1.029	0.018 ⁎⁎
LVESVi	1.021	1.008 – 1.034	0.001 ⁎⁎
LAVi	1.026	1.012 – 1.039	< 0.001 ⁎⁎
MR Severity	1.711	0.953 – 3.073	0.072
Likelihood of pHTN			
Low	-	-	-
Intermediate	2.223	1.126 – 4.390	0.021 ⁎⁎
High	5.626	2.189 – 14.461	< 0.001 ⁎⁎

⁎⁎ Denotes significant P values < 0.05.

Supplementary Table 1 – demonstrating the hazard ratio (HR) with 95% confidence intervals (CI) of individual variables on the risk of mortality. Variables include: age (HR 1.065; 95% CI 1.035-1.096; p<0.001), hypertension (HR 0.583; 95% CI 0.315-1.082; p=0.083), diabetes (HR 1.315; 95% CI 0.315-1.082; p=0.353), creatinine clearance (HR 0.981; 95% CI 0.971-0.991; p<0.001), left ventricular ejection fraction (LVEF) (HR 0.966; 95% CI 0.948-0.984; p<0.001), indexed left ventricular end-diastolic volume (LVEDVi) (HR 1.016; 95% CI 1.003-1.029; p=0.018), indexed left ventricular end-systolic volume (LVESVi) (HR 1.021; 95% CI 1.008-1.034; p=0.001), indexed left atrial volume (HR 1.026; 95% CI 1.012-1.039; p<0.001); MR severity (HR 1.711; 95% CI 0.953-3.073; p=0.072); intermediate likelihood of pulmonary hypertension (pHTN) (HR 2.223; 95% CI 1.126-4.390; p=0.021); high likelihood of pHTN (HR 5.626; 95% CI 2.189-14.461; p<0.001).

## 2 Experimental Design, Materials and Methods

A prospectively maintained National Institute for Cardiovascular Outcomes Research (NICOR) database maintains a record of all patients admitted with acute myocardial infarction, including type of infarction and procedure performed. This database was searched for patients fulfilling the inclusion criteria and found that a total of 1210 patients were admitted with a type-1 myocardial infarction (MI) between 2016 and 2017. Six researchers analysed 1000 consecutive records of patients who were treated by percutaneous coronary intervention (PCI). These records were cross referenced with the Queen Elizabeth Hospital Birmingham (QEHB) hospital records to identify those who had pre-discharge echocardiography reports. Duplicate records (patients attending for staged PCI or further MI within the timeframe were excluded). Hospital records of patients included in the study were further interrogated to identify:

• **Demographic data:** Age, sex, date of birth
• **Past history & risk factors:** Smoking status, family history of coronary artery disease, hypercholesterolaemia, hypertension, previous stroke, renal function, previous MI, previous coronary artery bypass grafting, previous PCI, previous MI, diabetes.
• **MI details:** Symptom onset date/time, type of MI (ST-elevation vs non-ST elevation), serum levels of B-type natiuretic peptide and high-sensitivity troponin T (Roche Cobas E170®) measured serially with recordings of admission (index) and peak values.
• **Procedure details:** Procedure date/time, angiographic findings, angioplasty details (including vessel treated, stent number, type, length) were obtained from an in-house purpose-built prospectively maintained database.
• **Medication:** Drug class prescribed.
• **Presentation:** Clinical heart failure (discharge letter reporting pulmonary oedema or record of patient requiring diuresis).
• **Echocardiogram:** Date/time, prior echocardiography (pre-dating MI), parameters assessing MR and chambers (full details below).
• **Outcomes:** Mortality data was obtained from the QEHB hospital records which are linked to centralised data from the Office of National Statistics. Date of death and time from MI to death was recorded.

### 2.1 Measurement of echocardiographic parameters

All included patients had echocardiography performed prospectively pre-discharge by technicians accredited by the British Society of Echocardiography using CX-50 machines (Philips medical systems, Amsterdam, Netherlands) and acquiring the minimum dataset [3].

• **MR severity assessment:** MR was categorized as none/trivial, mild, moderate, or severe according to established criteria [4]. Where possible, multiparametric assessment of MR was performed, as recommended [4], which includes the assessment of the following parameters:
  ○ Proximal isovelocity surface area (PISA) – measured (cm) with reduced aliasing velocity and increased penetration depth.
  ○ Vena contracta(VC) width – measured (cm) in the parasternal long axis view in a plane parallel to the mitral annulus.
  ○ Effective regurgitant orifice area (EROA) – derived by measurement of PISA using the flow convergence method, the aliasing velocity and the velocity-time-integral of the MR jet using continuous wave doppler in the apical 4-chamber view. The EROA was then calculated using the formula below:
    $$\text{EROA}=\frac{2\times \pi \times r^{2}\times \;V_{\text{Alias}}}{V_{max}}$$
    Whereby V_Alias_ represents the alising velocity at the radial distance (cm/s), V_max_ represents the peak velocity of the mitral regurgitant jet (cm/s) and r represents the PISA radius (cm).
  ○ Regurgitant volume (RVol) – calculated by multiplying the EROA by the velocity-time integral (cm) dervived from a continuous wave Doppler profile of the MR jet in the apical 4-chamber view.

• **Chamber assessment:** Left atrial (LA) and LV volumes were calculated using images acquired in the apical four and apical two chamber views. Images were acquired in end-systole (LA volume and LV end-systolic volume) and end-diastole (LV end-diastolic volume). The Simpson's biplane method was used to calculate volumes and values were indexed to the Mosteller calculation of body surface area [5]. LA dilatation was defined as a left atrial volume > 34ml/m^2^ after indexing to body surface area (LAVi) [6]. Left ventricular ejection fraction (LVEF) was calculated by the difference between LV end-diastolic and end-systolic volume, divided by the LV end-diastolic volume.
• **Mitral valve characteristics:** Colour flow doppler echocardiographic images of the regurgitant jet in the parasternal long-axis, apical 4-,3-and 2-chamber views were analysed subjectively to obtain data regarding regurgitant jet direction. Information pertaining to the presence of mitral annular calcification and leaflet thickness were obtained from the echocardiographic reports.

### 2.2 Interventional treatment methods

All study patients underwent PCI and were treated according to European guidelines on the management of AMI [7,8]. This includes medical therapy with loading and maintenance doses of aspirin and P2Y12 receptor blocker, beta blockers, statins and angiotensin converting enzyme inhibitors [7,8]. Coronary lesions were graded by visual assessment of angiographic stenosis by the consultant interventional cardiologist treating the patient. Severe disease was defined as >50% stenosis of the left main stem and >75% stenosis of all other coronary vessels.

### 2.3 Statistical methods

Normal and non-normally distributed continuous variables are expressed as mean + standard deviation or median with interquartile range and compared using a Student's two-tailed T test or Mann-Whitney U test respectively. Categorical variables were compared using a chi squared test. A P value of < 0.05 was considered significant. Factors influencing survival were assessed by a univariate Cox regression model and analysed using SPSS software version 23.0 (SPSS, Inc, Chicago, IL, USA).

## Ethics Statement

This study was a registered retrospective observational audit performed in compliance with local clinical and research governance principles.

## Credit Author Statement

**Harish Sharma:** Conceptualization, Data collection and analysis, Writing manuscript; **Ashwin Radhakrishnan:** Conceptualization, Data collection, Manuscript review; **Peter Nightingale:** Statistical analysis; **Samuel Brown:** Data collection; **John May:** Data collection; **Kieran O'Connor:** Data collection, **Iqra Shakeel:** Data collection; **Nawal Zia:** Data collection; **Sagar N. Doshi:** Manuscript review; **Jonathan N. Townend:** Manuscript review; **Saul G. Myerson:** Manuscript review; **Paulus Kirchhof:** Manuscript review; **Peter F Ludman:** Data sourcing and manuscript review; **M. Adnan Nadir:** Conceptualization, project supervision and manuscript review; **Richard P. Steeds:** Conceptualization, project supervision and manuscript review.

## Declaration of Competing Interest

The authors declare that they have no known competing financial interests or personal relationships which have or could be perceived to have influenced the work reported in this article.

Prof Myerson and Dr Sharma are supported by the National Institute for Health Research (NIHR) Oxford Biomedical Research Centre and Dr Sharma was also funded by the Birmingham Health Partners Research Starter Fellowship. This work was partially supported by European Union BigData@Heart (grant agreement EU IMI 116074 to Prof Kirchhof), 10.13039/501100000274British Heart Foundation (FS/13/43/30324, PG/17/30/32961, PG/20/22/35093 and AA/18/2/34218 to Prof Kirchhof), German Centre for Cardiovascular Research supported by the German Ministry of Education and Research (DZHK, via a grant to AFNET to Prof Kirchhof) and Leducq Foundation to Prof Kirchhof.

Disclosures: Prof Kirchhof receives research support for basic, translational, and clinical research projects from European Union, British Heart Foundation, Leducq Foundation, Medical Research Council (UK), and German Centre for Cardiovascular Research, from several drug and device companies active in atrial fibrillation, and has received honoraria from several such companies in the past, but not in the last three years. Prof Kirchhof is listed as inventor on two patents held by University of Birmingham (Atrial Fibrillation Therapy WO 2015140571, Markers for Atrial Fibrillation WO 20160.

## Appendix Supplementary Materials

**Supplementary Table 1** table demonstrating univariate Cox proportional hazards regression for mortality. Image, application 1

## Footnotes

Supplementary material associated with this article can be found in the online version at doi:10.1016/j.dib.2021.107451.
